# Electromechanical Behaviors of Graphene Reinforced Polymer Composites: A Review

**DOI:** 10.3390/ma13030528

**Published:** 2020-01-22

**Authors:** Chuang Feng, Dong Zhu, Yu Wang, Sujing Jin

**Affiliations:** 1 College of Civil Engineering, Nanjing Tech University, Nanjing 211816, China; 2 Zhejiang Scientific Research Institute of Transport, Hangzhou 311305, China; zhengjz@zjjtkyy.com; 3 School of Engineering, RMIT University, Melbourne 3083, Australia; s3415279@student.rmit.edu.au

**Keywords:** electromechanical behavior, graphene, polymer composites

## Abstract

Graphene (including its derivatives)-reinforced polymer composites (GRPCs) have been drawing tremendous attention from academic and industrial communities for developing smart materials and structures. Such interest stems from the excellent combination of the mechanical and electrical properties of these composites while keeping the beneficial intrinsic attributes of the polymers, including flexibility, easy processability, low cost and good biological and chemical compatibility. The electromechanical performances of these GRPCs are of great importance for the design and optimization of engineering structures and components. Extensive work has been devoted to this topic. This paper reviews the recent studies on the electromechanical behaviors of GRPCs. First the methods and techniques to manufacture graphene and GRPCs are introduced, in which the pros and cons of each method are discussed. Then the experimental examination and theoretical modeling on the electromechanical behaviors of the nanocomposites are presented and discussed.

## 1. Introduction

The two-dimensional (2D) graphene with a honeycomb structure has demonstrated excellent mechanical and physical properties. For example, its Young’s modulus, thermal conductivity and electrical conductivity can reach as high as 1 TPa, 5000 W·m^−1^·K^−1^ and 6000 S·cm^−1^, respectively [1,2,3]. Various techniques and methods have been developed to produce graphene fillers, such as mechanical exfoliation [4,5], liquid phase exfoliation [6,7], electrochemical exfoliation [8,9], reduction of graphene oxide [10,11], chemical vapor deposition [12,13] and epitaxial growth [14,15]. Although graphene possesses the abovementioned excellent properties, they need to be incorporated into a continuous phase, such as a polymer, to cater for different applications. Polymer matrix can bond the graphene fillers together while showing elastic, electrical and flexible properties that are favorable for lots of engineering applications. A wide range of polymers involving elastomers [16], block copolymers [17], epoxy [18], thermoplastics [19,20] and hydro/aerogels [21,22] have been adopted to manufacture graphene-reinforced polymer composites (GRPCs). In addition to monolayer graphene, its derivatives, including graphene nanoplatelet (GNP), graphene oxide (GO) and reduced graphene oxide (rGO), are also widely used to manufacture high-performance GRPCs due to their comparable material properties with moderate cost.

When graphene fillers are distributed in a polymer matrix, both theoretical and experimental work has evidenced significant enhancement on the mechanical and electrical properties of GRPCs. Rafiee et al. [5] observed that the addition of 0.1 wt % GNP into polymer can increase the modulus by 52%.

Zhao et al.’s [23] experiments found that the modulus of graphene nanosheet (GN) reinforced polyvinyl alcohol (PVA) can reach up to 10-folds of pristine PVA. The molecular dynamics (MDs) simulation developed by Sun et al. [24,25,26] and Rahman et al. [27,28,29,30] exhibited significant increases in the mechanical properties of GRPCs. The micromechanics modeling work [31,32,33,34] also indicated graphene fillers’ prominent enhancement on the mechanical properties of GRPCs. More work on mechanical properties can be found in [35,36,37,38,39,40]. In addition to the enhancement on mechanical properties, graphene can also greatly improve the electrical property of the insulating polymer matrix. Imran and Shivakumar [41] prepared GRPCs and found that approximately 1.0 wt % graphene can enhance the electrical conductivity from 4.3 × 10^−15^ S·m^–1^ to 2.6 × 10^−6^ S·m^–1^. Ravindran et al. [42] experimentally examined the effects of surface area and dimensions of graphene on the electrical conductivity and dielectric permittivity of the GRPCs. They found larger sized graphene fillers with greater surface area have better reinforcing effects compared to their counterparts with smaller size and surface area. Fan et al.’s [43] experiments examined the dielectric permittivity of graphene-reinforced polyvinylidene fluoride (PVDF) composites. The dielectric constant can reach as high as 7940 at 100 Hz of alternating current (AC) frequency by dispersing 1.77 vol % of graphene. Significant increase in the dielectric property of GRPCs, i.e., five times larger than that of pure polymer, was observed by Chen et al.’s [44] experiments. By adding 1.01 vol % GNPs, He et al. [45] found that the dielectric constant of their fabricated PVDF nanocomposites can reach up to 200 at 1000 Hz (AC frequency) and 2700 at 100 Hz, respectively. Apart from experimental studies, Xia et al. [46] developed the effective-medium theory (EMT) model to predict the electrical properties of GRPCs. The model, which was validated by experimental results, observed obvious enhancement in the electrical conductivity and dielectric permittivity of GRPCs. Wang et al. [47,48,49] used the EMT and analyzed the behaviors of GRPC structures. Their results advised that the behaviors of the GRPC structures can be actively tuned by adjusting the attributes of the external electrical field. More studies on electrical properties can be referred to [38,50,51,52,53,54,55].

The electromechanical behaviors of GRPCs, which denotes the performance of the electrical properties of the composites subjected to mechanical deformation, have attracted tremendous attention due to their potential in developing smart materials and structures. This review will conduct a survey of the literatures on the coupling of the mechanical and electrical behaviors of GRPCs from the perspectives of experiment and theoretical modeling. First, we review the preparation of graphene reinforcements and approaches to prepare GRPCs. The experimental examination and theoretical modeling on the electromechanical behaviors of the GRPCs are then discussed.

## 2. Graphene-Reinforced Polymer Composites

### 2.1. Graphene

There are six major methods to manufacture graphene, which includes mechanical exfoliation, liquid phase exfoliation, electrochemical exfoliation, chemical vapor deposition, the reduction of Graphene Oxide and epitaxial growth. Each method can be evaluated by five factors, i.e., quality, cost, scalability, purity and yield are used to evaluate each method. Table 1 shows the comparisons of the six methods in terms of the five factors. Details of each method are also discussed following the table.

(a) Mechanical Exfoliation

**Figure 1:**
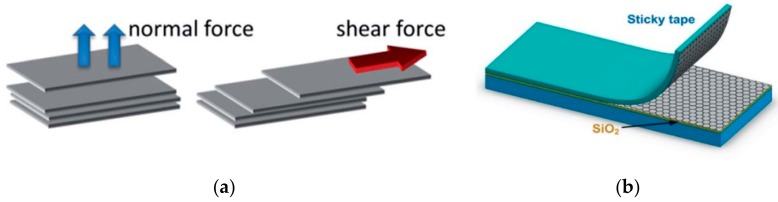
(**a**) Two routes for mechanical exfoliation; (**b**) Micromechanical cleavage for graphene production. Reproduced with permission from the authors of [56,57]. Copyright 2012, 2015, Elsevier and Royal Society of Chemistry.

Mechanical exfoliation is a simple and low-cost mechanical method to obtain graphene with high quality. As shown in Figure 1a, there are two routes for mechanical exfoliation [56]. One can use normal force to overcome the van der Waals attraction between neighboring layers to exfoliate. This micromechanical cleavage method, which is carried out by repeated tape peeling, as illustrated in Figure 1 [57]. It is also the one to firstly obtain monolayer graphene by the Nobel Prize winner in 2010. Another method is to exert shear force to exfoliate, which can be achieved by roll-mill. Due to its intrinsic attributes and limitations, mechanical exfoliation is very difficult to scale up the production. Therefore, this method is usually adopted to gain graphene for lab research use only, and is not suitable for any industrial application purpose.

(b) Liquid Phase Exfoliation

**Figure 2:**
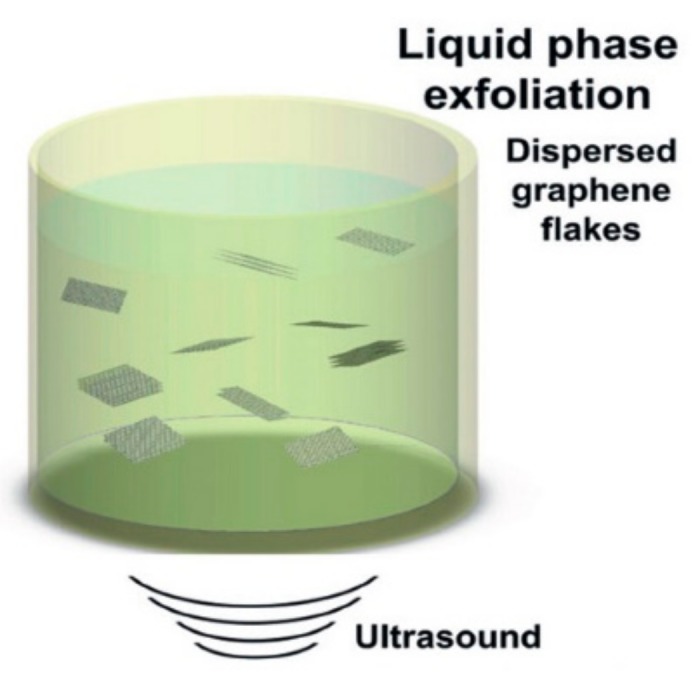
Liquid phase exfoliation for graphene production. Reproduced with permission from the authors of [57]. Copyright 2012, Elsevier.

Liquid phase exfoliation for the production of graphene has three steps, i.e., dispersion, exfoliation and purification. First of all, solvent is introduced to reduce the potential energy between adjacent layers of graphite. Then two major methods, sonication and high shear, are used for exfoliation. Figure 2 shows the process of sonication [57]. This process is beneficial to produce nanocomposites with a high concentration of graphene. However, sonication also has some disadvantages, such as high time and energy consumption. It also introduces defects into graphene. In contrast, the high-shear method can produce large amounts of defect-free graphene dispersion within a shorter time period. For the last step, different sizes of graphene are separated by applying different centrifugation rates. The lateral size of graphene produced by centrifugation is usually small. This makes the graphene produced by this method a less efficient reinforcing filler for high-performance composites [58].

(c) Electrochemical Exfoliation

**Figure 3:**
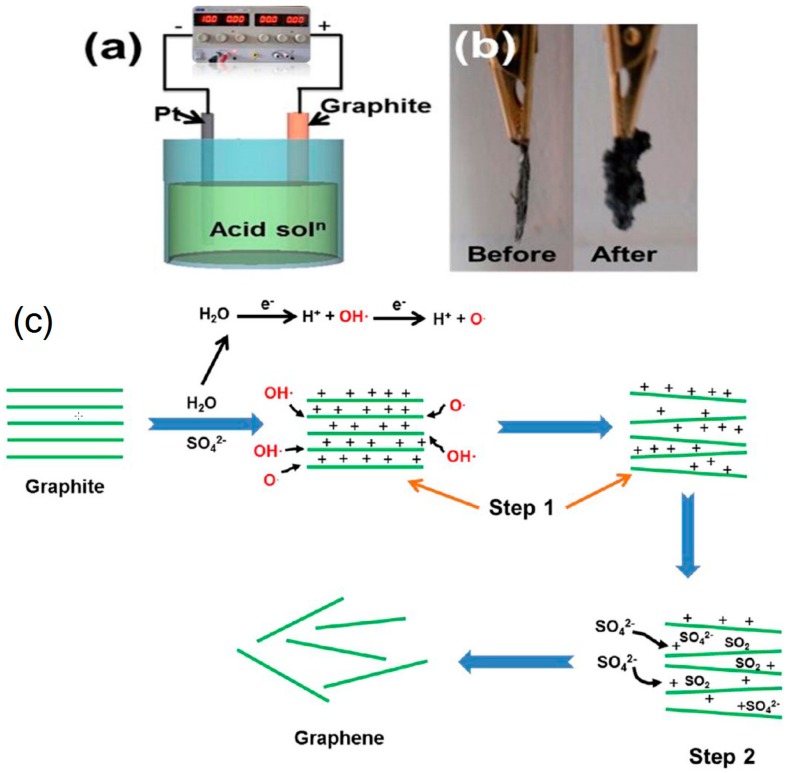
Electrochemical exfoliation for graphene production. (**a**) Electrochemical exfoliation; (**b**) Samples before and after exfoliation; (**c**) Structures of graphene before and after electrochemical exfoliation. Reproduced with permission from [63]. Copyright 2013, American Chemical Society.

This is a method that uses a conductive solution as an electrolyte and graphite-based materials as the sacrificing electrode, respectively. This method involves anodic oxidation or cathodic exfoliation, as shown in Figure 3. During the process, ions from the conductive solution are intercalated into graphite with the assistance of electrical potential. For the anodic oxidation method, the graphite rods work as an anode and the ions intercalate with graphite to produce graphene. The graphene prepared by this method has oxygen functional groups that can restrict its electrical conductivity and application. Moreover, there are also structural defects on graphene sheets during anodic oxidation reactions [8,59]. In contrast, cathodic exfoliation can produce high-quality graphene with fewer layers and low level of oxygen [60,61,62]. Electrochemical exfoliation is recognized as a promising method for the large scale manufacture of graphene.

(d) Chemical Vapor Deposition

**Figure 4:**
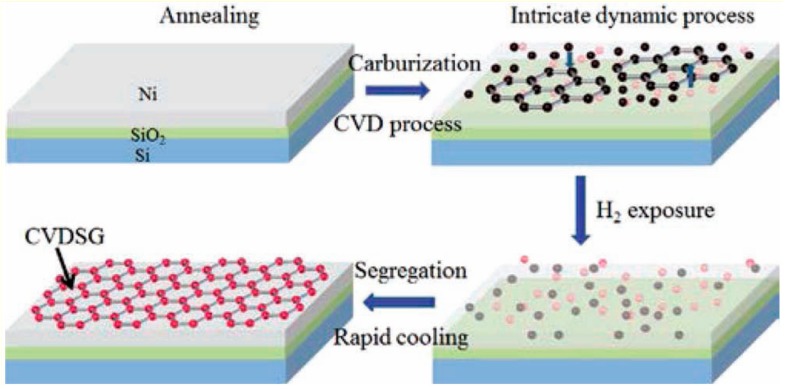
Chemical vapor deposition for graphene production. Reproduced with permission from [64]. Copyright 2012, John Wiley & Sons.

Chemical vapor deposition (CVD) is a facile method for the large-scale manufacture of graphene with high-quality. Carbon atoms firstly are deposited and nucleated on a substrate, and then are grown into large domains, as shown in Figure 4 [64]. A major problem of CVD is the removal of monolayer graphene from the substrate, which may introduce defects and wrinkles. Another challenge for CVD is the requirement of high energy. Despite the existing challenges, CVD is still recognized as another promising technique for large-scale production of graphene.

(e) Reduction of Graphene Oxide

Graphene can also be obtained from GO by eliminating oxygen functional groups. Two main techniques, i.e., chemical and nonchemical reductions, are used. For chemical reduction, it consists of the exfoliation of GO and the subsequent reduction of the exfoliated single layer GO. During the process, GO is reduced in colloidal suspension with various agents. For nonchemical reduction, it includes several methods, including thermal reduction, electrochemical reduction, photocatalytic reduction, sun light irradiation and supercritical fluid conversion.

(f) Epitaxial Growth

**Figure 5:**
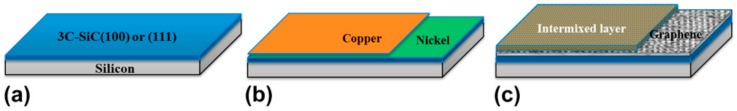
Epitaxial growth for graphene production. Reproduced with permission from [65]. (**a**) Starting substrate; (**b**) Deposit catalyst layer of Ni or Ni and Cu on starting substrate; (**c**) Anneal for the formation of graphene covered with intermixed layer. Copyright 2015, Cambridge University Press.

Figure 5 [65] shows the epitaxial growth process for graphene production. Carbon atoms are firstly self-assembled into a honeycomb lattice on silicon carbide (SiC) materials. The electronic mobility of graphene films formed can be as high as 2000 cm^2^·V^−1^·s^−1^ [66]. Graphene grown on an electrically insulating substrate by epitaxial growth is ideal for the application of graphene in electronic devices subjected to high temperature and frequency. Although the epitaxial growth method is low-cost, it is not applicable for preparing nanocomposites reinforced with graphene.

### 2.2. Polymer Matrix

Different kinds of polymer matrices have been used for preparing GRPCs, including highly stretchable elastomers with long chains lightly crosslinked [16,67,68], nonchemical crosslinked thermoplastic polymer [19,20,69], heavily crosslinked epoxy resins [18,70,71], block copolymers with intrinsic heterogeneous properties [17,72,73], and porous structured hydrogels or aerogels [21,22,74]. Each kind of polymer has pros and cons. For example, highly stretchable elastomers can elongate up to 1000% of their length, but the strength, stiffness and electrical conductivity of such composites are low, which restricts their potential applications [16]. For thermoplastics, they have high strength and stiffness, but no electrical conductivities [19,75]. Epoxy resins have excellent strength, stiffness and electrical properties. However, their brittleness and poor flame resistance limited their application in industry [70,71].

Block copolymers are used as a template to precisely locate the fillers’ position in the composite due to the advantage of their covalently-bonded blocks [76,77]. With microporous and mesoporous structure, hydrogel and aerogel showed their advantages in electronic materials [22,78]. Table 2 tabulates a series of polymer matrices together with the graphene type and preparation method used to develop graphene nanocomposites.

### 2.3. Graphene Reinforced Polymer Composites

As demonstrated in Table 2, several methods have been used to prepare GRPCs. The methods are introduced and discussed in the following.

(a) Solution Mixing

**Figure 6:**
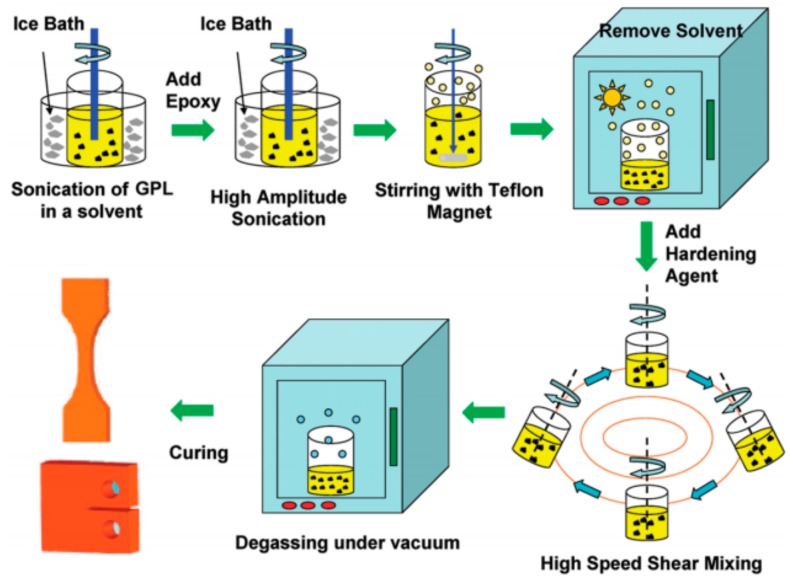
Solution mixing for manufacture of graphene-reinforced polymer composites (GRPCs). Reproduced with permission from the authors of [5]. Copyright 2009, American Chemical Society.

Solution mixing is a process by dispersing graphene fillers into polymer solutions through ultrasonication and shearing according to the solubility of the fillers in the solvents. Figure 6 shows the process of dispersing graphene fillers into polymer solutions [5]. Many polymers, such as PVA, PMMA, PCL, PLA and PU, can be used to produce GRPCs with a low percolation threshold by using solution mixing. However, there are also some disadvantages with this method. For example, toxic solvents are usually introduced while dispersing graphene fillers into polymers [105,106]. Removing the introduced toxic solvents restricts the largescale production of the composites.

(b) Melt Blending

Polymers can be melted and mixed with graphene fillers to produce composites as the temperature increases. For example, graphene fillers can be blended with Polypropylene (PP) first and then migrated to Polyethylene (PE) with further melt blending, which formed graphene/PE/PP nanocomposite. This approach is normally used to produce thermoplastic polymer composite in industry due to its fast, cost effective, simple and high yield strength attributes. Polymers that can use this method to produce graphene/polymer composites include PLA [107], polyethylene terephthalate (PET) [104], isotactic polypropylene (iPP) [69] and Polycarbonate (PC) [95].

(c) In Situ Polymerization

**Figure 7:**
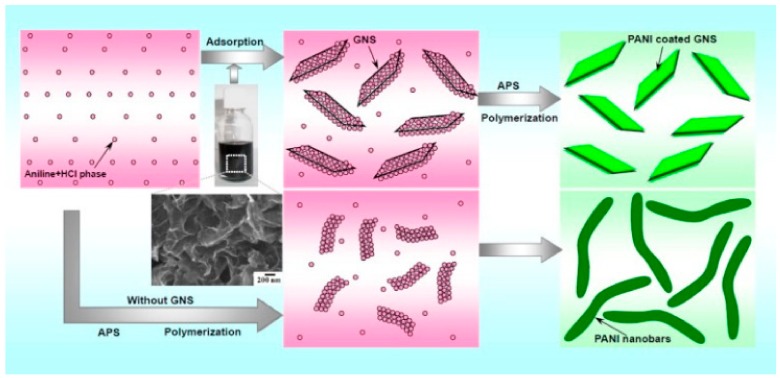
In-situ polymerization for manufacture of GRPCs. Reproduced with permission from [108]. Copyright 2010, Elsevier.

Graphene fillers can be well dispersed in polymers by using in-situ polymerization without any pre-exfoliation step. Strong interactions occur between the polymer matrix and the graphene fillers during the polymerization process. GRPCs prepared by this method showed improved mechanical property with a lower percolation threshold compared to solution mixing and melt blending. Figure 7 [108] shows the mixture of monomer solution, graphene and catalyst for in-situ polymerization. Epoxy resins [109], nylon-6 [98], silicone [99], PS [100,101], polyvinyl chloride acetate (PVCA) [110] and PANI [108] are polymers suitable for the in-situ polymerization method. However, due to high energy consumption, in-situ polymerization is not suitable for the large-scale production of GRPCs.

(d) Layer-by-Layer Assembly

**Figure 8:**
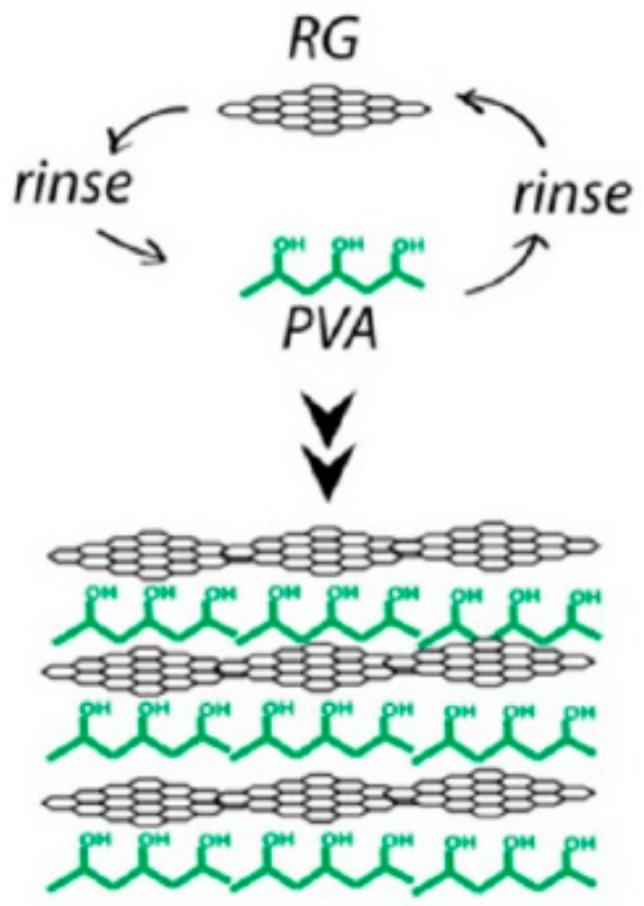
Layer-by-layer assemble for manufacture of GRPCs. Reproduced with permission from [111]. Copyright 2013, American Chemical Society.

This is a method that can precisely manipulate the distribution of graphene fillers in the composite. Hierarchical nanostructured GRPC can be produced by changing anionic and cationic phases. Alternating the order of deposition, the composites can be manufactured for various applications, such as batteries, membranes and super capacitors. Polymers, including PDDA, PAH and PVA, have been used to develop the nanocomposites by using layer-by-layer assembly. Figure 8 [111] shows the layer-by-layer assembly, which is achieved by immersing substrate in PVA solutions with reduced graphene dispersion in a prescribed sequence, and rinsing intermittently with water. Layer-by-layer assembly can be used for preparing GRPCs with controlled nanoscale architectures, mechanical property and thickness.

## 3. Electromechanical Behaviors

### 3.1. Experiments

Mechanical deformation (i.e., stretching, compressing, bending, etc.) can vary the electrical properties of GRPCs due to the variation in the molecular structures and orientation of the graphene fillers. Table 3 summarized the experimental studies on the mechanical and electrical properties of GRPCs, following which some experimental studies are discussed in detail.

**Figure 9:**
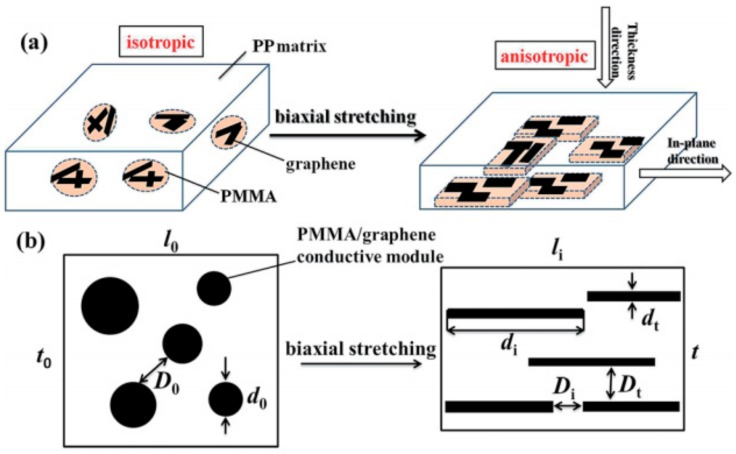
(**a**) Morphology transformation of GRPCs during biaxial stretching; (**b**) Sectional view of (a). Reproduced with permission from [121]. Copyright 2017, The Royal Society of Chemistry.

**Figure 10:**
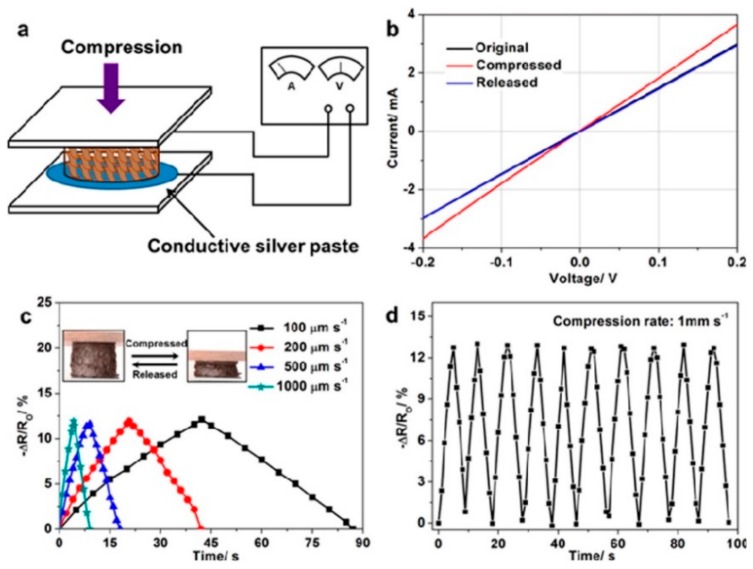
Electromechanical behaviors of graphene aerogel/plasma desorption ionization mass spectrometry (PDMS) composites. (**a**) Setup for testing; (**b**) Current-Voltage curves; (**c**) Resistance change with strain at different compression rates; (**d**) Resistance change with at fixed compression rate. Reproduced with permission from [138]. Copyright 2014, American Chemical Society.

By sequential biaxial stretching as shown in Figure 9, You et al. [121] proposed an efficient approach to orientate fillers in the in-plane direction, which significantly enhanced the electrical conductivity in this direction. By using the CVD technique, Chen et al. [134] fabricated three-dimensional graphene foam-reinforced PDMS composites and investigated the variation of the electrical resistance with stretching cycles and strain. With the increase of the stretching cycles, the resistance first increases and then becomes stable. For example, from the sixth cycles, the resistance keeps constant when the strain is released, indicating the stable electromechanical performances of the GRPCs. Hu et al. [138] proposed an infiltration-evaporation-curing method to fabricate hybrid structure, which consists of compressible graphene aerogel as reinforcing fillers, and PDMS as the polymer matrix. As shown in Figure 10, this hybrid structure showed excellent electromechanical stability during repeated compress process (as shown in Figure 10d).

**Figure 11:**
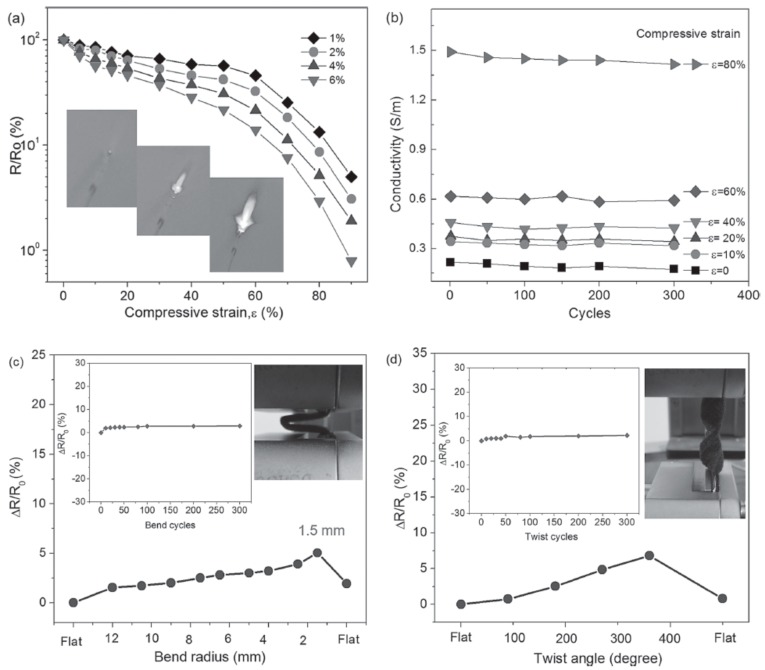
Resistance and conductivity of graphene/polyurethane (PU) composites. (**a**) Resistance change with strain for different graphene concentrations; (**b**) Electrical conductivity of composites with 6% graphene after compression-release cycles; (**c**) Resistance change with bending radius with 6% graphene; (**d**) Resistance change with twist angle with 6% graphene. Reproduced with permission from [119]. Copyright 2013, John Wiley & Sons.

**Figure 12:**
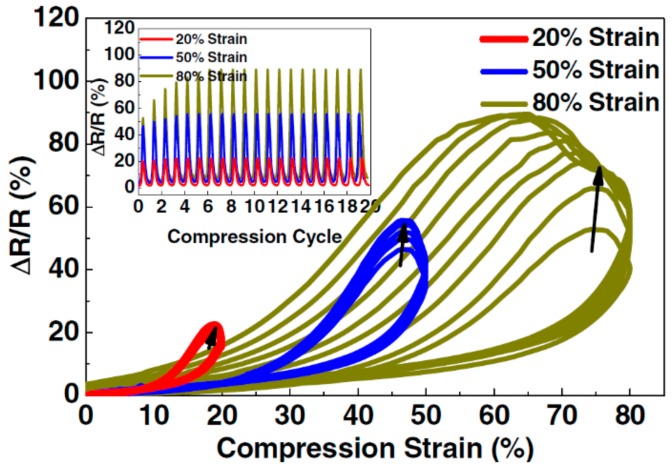
Electrical resistance of graphene aerogel/PDMS composites subjected to compression strain. Reproduced with permission from [114]. Copyright 2015, Elsevier.

Wu and his colleagues [119] assembled graphene nanosheets on a three-dimensional PU skeleton to form GRPCs. Figure 11 demonstrates the electrical resistance change and electrical conductivity of the composites under mechanical deformation. The resistance decreases linearly when the strain is approximately less than 60%. However, when the strain further increases to be greater than 70%, the resistance decreases exponentially. After 300 cycles at fixed strain, the electromechanical performance of the composites becomes stable with negligible variation in the resistance change. A similar trend can also be found for the influences of bending and twisting on the resistance change. Zhang et al. [114] fabricated 3D graphene aerogel/PDMS composites and also observed outstanding electromechanical properties under cyclic compressive strains (as shown in Figure 12).

**Figure 13:**
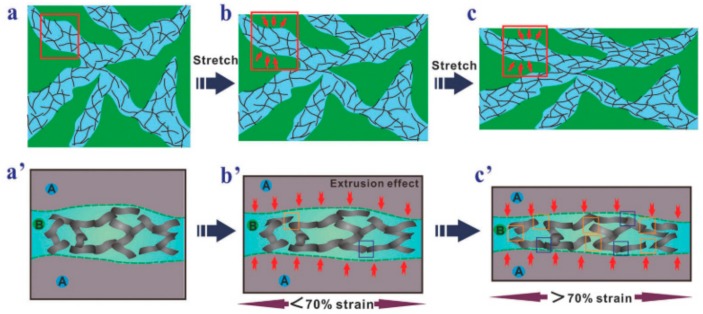
Formation of the conductive network in GRPCs subjected to stretching. (**a**) State without stretching; (**b**) State with stretching being less than 70%; (**c**) State with stretching being more than 70%; (**a’**), (**b’**) and (**c’**) are the corresponding magnified images of the region in the rectangular shapes. Reproduced with permission from the authors of [136]. They Royal Society of Chemistry.

Lin et al. [136] fabricated a sensitive strain sensor which is based on graphene-reinforced rubber composite. Figure 13 presents the formation of the conductive network in the composites when subjected to stretching. It is found that the nanocomposite-based sensors exhibited a high stretchability, sensitivity (i.e., the gauge factor can reach up to 82.5) and good reproducibility (up to 300 cycles) when subjected to the cyclic tensile test.

**Figure 14:**
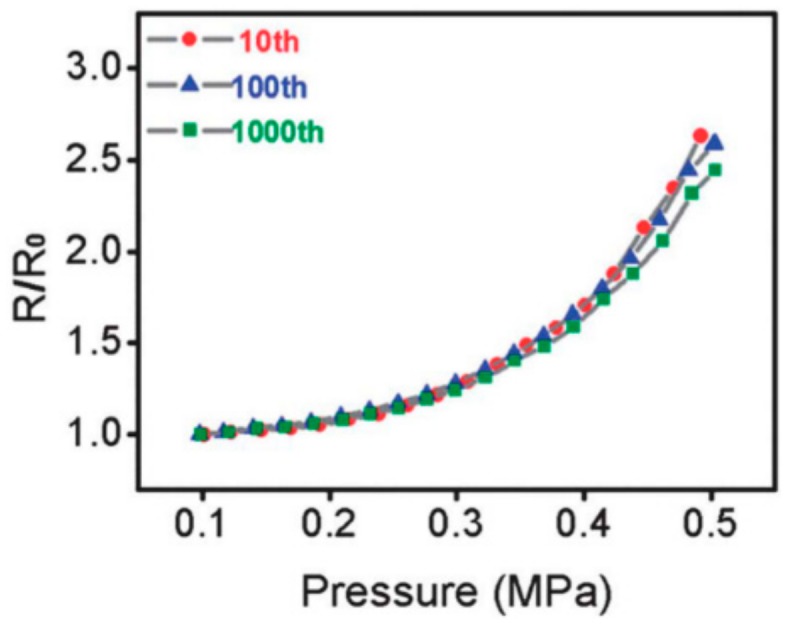
Resistance change versus pressure under cyclic test. Reproduced with permission from [139]. Copyright 2015, Elsevier.

Xu et al. [128] fabricated graphene-reinforced elastomer sensors with changeable properties. It is found that these sensors are sensitive to the out-of-plane bending, while they are not sensitive to the in-plane stretching. Wu et al. [132] reported a new type of strain sensors with vertical graphene fillers sandwiched between two neighboring PDMS layers. Such sensors have high stretchability (~ 120%) and high sensitivity. Hou et al. [139] investigated the piezoresistivity of PDMS nanocomposites reinforced with functionalized graphene as a conductive filler. As seen from Figure 14, the resistance increases exponentially with pressure when the composites are under uniaxial compression. After 1000 load-release cycles, the curves remain nearly unchanged, indicating excellent durability and electromechanical stability.

**Figure 15:**
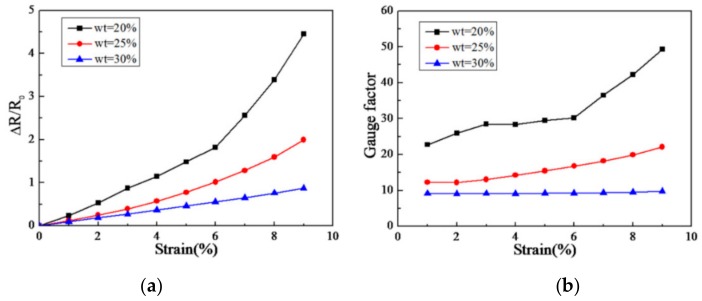
Resistance change with strain for different concentrations. (**a**) Resistance change with strain; (**b**) Variation of gauge factor with strain. Reproduced with permission from [133]. Copyright 2016, AIP Publishing.

Choi et al. [133] fabricated a strain sensor using PDMS that was reinforced with uniformly distributed graphene flakes. As shown in Figure 15, for greater graphene concentration, i.e., 30 wt %, the resistance change of the composites demonstrates a linear response with respect to the strain. For smaller concentrations, i.e., 20 wt % and 25 wt %, the gauge factor increases with the strain while it keeps constant when the concentration increases to 30 wt %. Generally, it can be concluded that the decrease of the filler concentration increases the relative electrical resistance change with the strain. Larger filler concentration enables the graphene-reinforced composites to have more stable gauge factors compared to their counterparts with smaller filler concentration.

**Figure 16:**
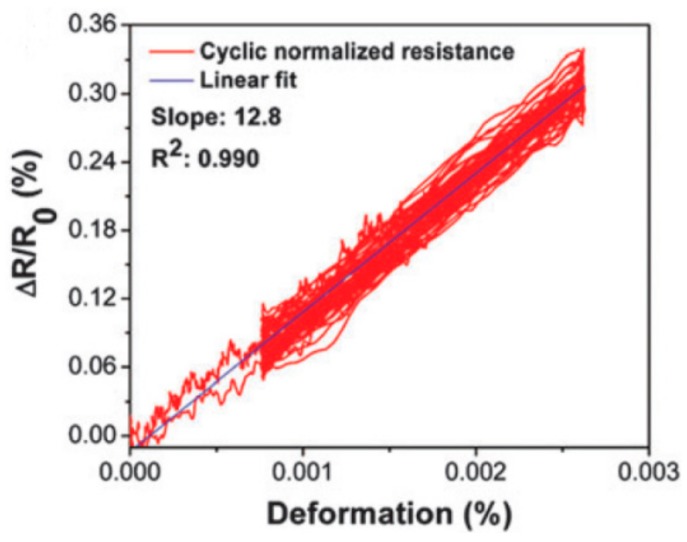
Resistance–deformation relationship of graphene/epoxy composites. Reproduced with the permission from the authors of [140]. Copyright 2016. The Royal Society of Chemistry.

Tung et al. [140] combined epoxy polymer and graphene to develop composites for sensors. Figure 16 shows the linear electrical resistance–deformation relationship. The electromechanical performance of the composites, which are subjected to static and dynamic deformation, demonstrated fast response (20 s) and excellent sensitivity (i.e., gauge factor of 12.8). Such sensors with outstanding electromechanical properties can be applied as mechanical strain sensors for real-time monitoring of health of structures.

**Figure 17:**
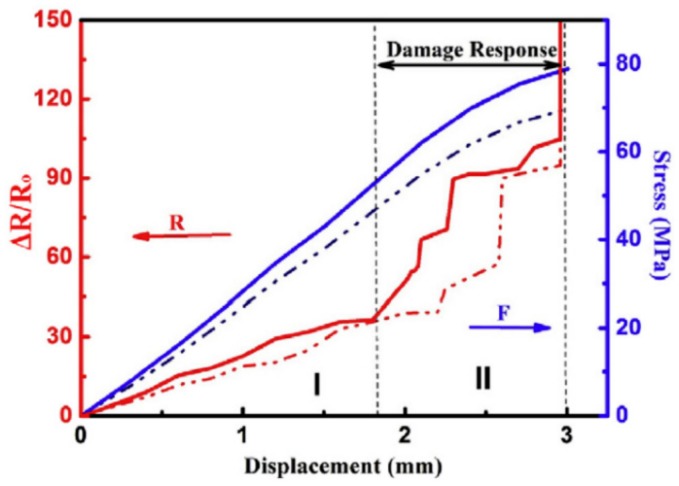
Electromechanical behaviors of GN/epoxy composites. Solid and dotted lines denote two samples. Reproduced with permission from [127]. Copyright 2016, Elsevier.

Zha et al. [127] developed sensors with high sensitivity, embedding three-dimensional graphene network into epoxy matrix. Seen from Figure 17, the resistance changes linearly at the beginning and then has nonlinear, ladder-shaped growth, indicating the potential application of such strain sensors in monitoring the irreversible deformation and damage in engineering structures.

**Figure 18:**
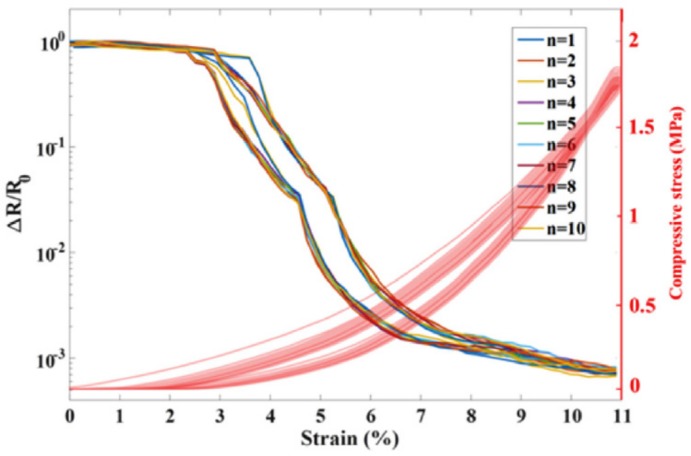
Electromechanical behaviors of GO/PLA/PEG composites for ten cycles. Reproduced with permission from [118]. Copyright 2018, Elsevier.

Scaffaro et al. [118] distributed GO into PLA- PEG and prepared a piezoresistive sensor. Figure 18 shows the electromechanical behaviors (resistance–strain curve) for ten cycles with strain up to 11%. It is demonstrated that the electrical properties, which stem from the addition of GO fillers, are sensitive to the mechanical deformations. For example, responsivities of 35 mA/MPa and 19 mA/MPa are observed for pressure ranges 0.6 to 8.5 MPa and 8.5 to 25 MPa, respectively.

**Figure 19:**
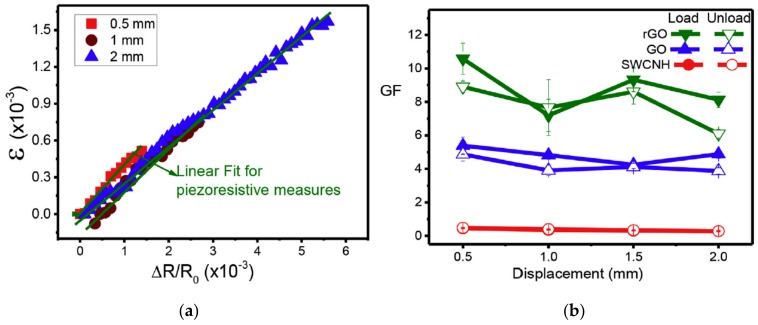
Electromechanical performances of rGO/PVDF composites. (**a**) resistance–strain relationship of the composites subjected to different deformations; (**b**) Gauge factor of the composites. Reproduced with permission from [120]. Copyright 2017, Elsevier.

**Figure 20:**
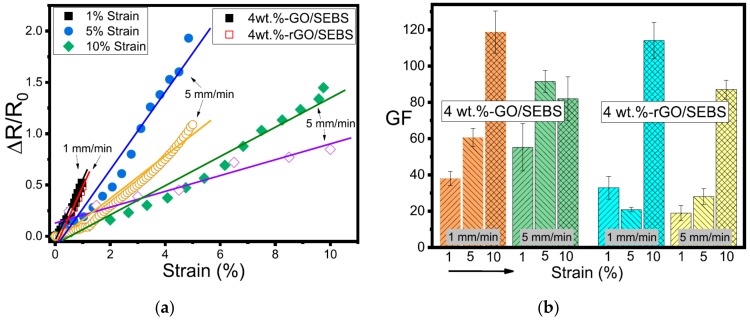
Electromechanical performances of GO/rGO SEBS polymer composites. (**a**) Relative electrical resistance change of the composites with strain; (**b**) GF of GO/SEBS and rGO/SEBS composites with 4 wt % filler content. Reproduced with permission from [89]. Copyright 2019, American Chemical Society.

Costa et al. [120] investigated the electromechanical behaviors of PVDF composites reinforced by different carbon nanofillers. As observed from Figure 19, linear fit is found for the resistance–strain relationship of the rGO-reinforced PVDF composites when subjected to different deformations. In addition, the rGO-reinforced composites have the highest gauge factor for all deformations. Recently, Costa et al. [89] developed GO and rGO reinforced styrene−ethylene−butylene−styrene (SEBS) composites and investigated the electrical and electromechanical properties of the composites. As demonstrated in Figure 20, linear fitting between the relative electrical resistance and the strain is obtained. A higher increasing rate of the relative electrical resistance change with stretching strain is found for the composites with smaller filler concentration. The GO/SEBS and rGO/SEBS composites show excellent electromechanical performances with gauge factors being up to 120, which can be used as a promising material candidate for strain sensor applications.

**Figure 21:**
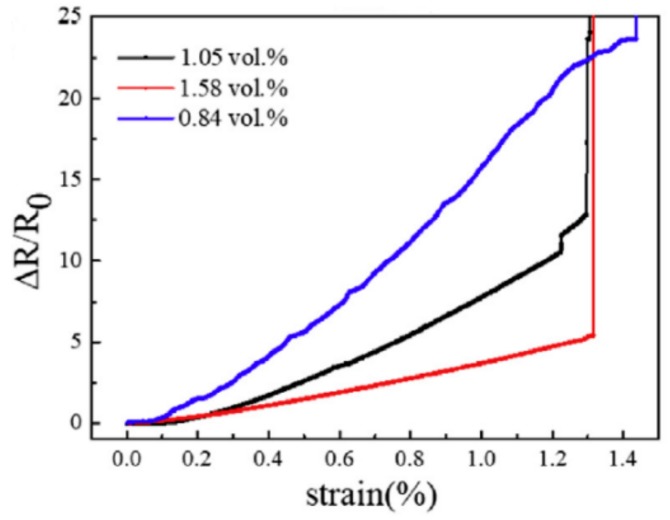
Response of GNP/epoxy nanocomposites to strain for different filler concentrations. Reproduced with permission from [123]. Copyright 2018, Elsevier.

Boland et al. [117] added graphene fillers into a lightly crosslinked polysilicone to prepare nanocomposites with substantially improved electromechanical behaviors. For example, the manufactured nanocomposite demonstrated a temporal relaxation of resistance after deformation and the linear variation of resistivity with strain. Such nanocomposites can be used to fabricate sensitive electromechanical sensors with gauge factors greater than 500. Lu et al. [123] prepared GNP-reinforced epoxy flexible sensors with relatively low percolation threshold. These flexible sensors can be applied to detect the damage and deformation of engineering structures with controllable performances. Figure 21 shows the response of the nanocomposites reinforced with GNPs at different concentrations. As the GNP concentration increases, the linear growth of the resistance change drops, while the linear tendency is enhanced. Such nanocomposite sensors demonstrated a great potential application in the damage monitoring of structural health.

**Figure 22:**
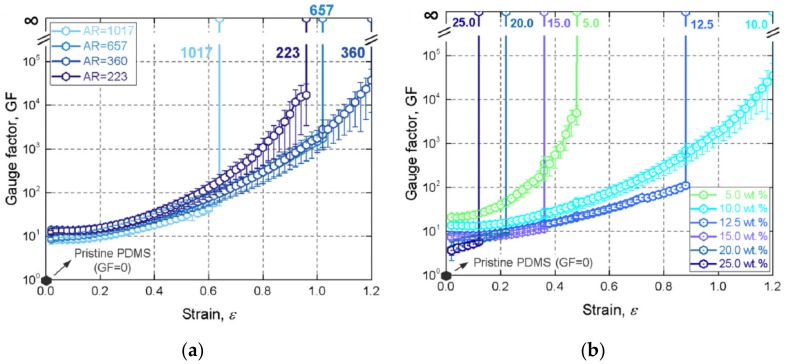
Gauge factor versus strain for nanocomposites with different graphene aspect ratios and concentrations. (**a**) Different GNF aspect ratios; (**b**) Different GNF concentrations. Reproduced with permission from [135]. Copyright 2018, Elsevier.

**Figure 23:**
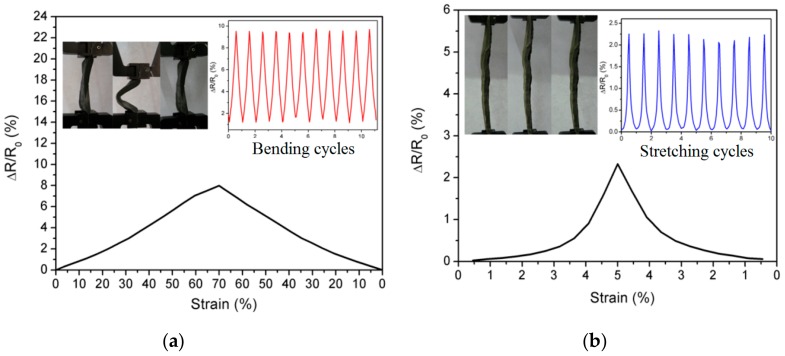

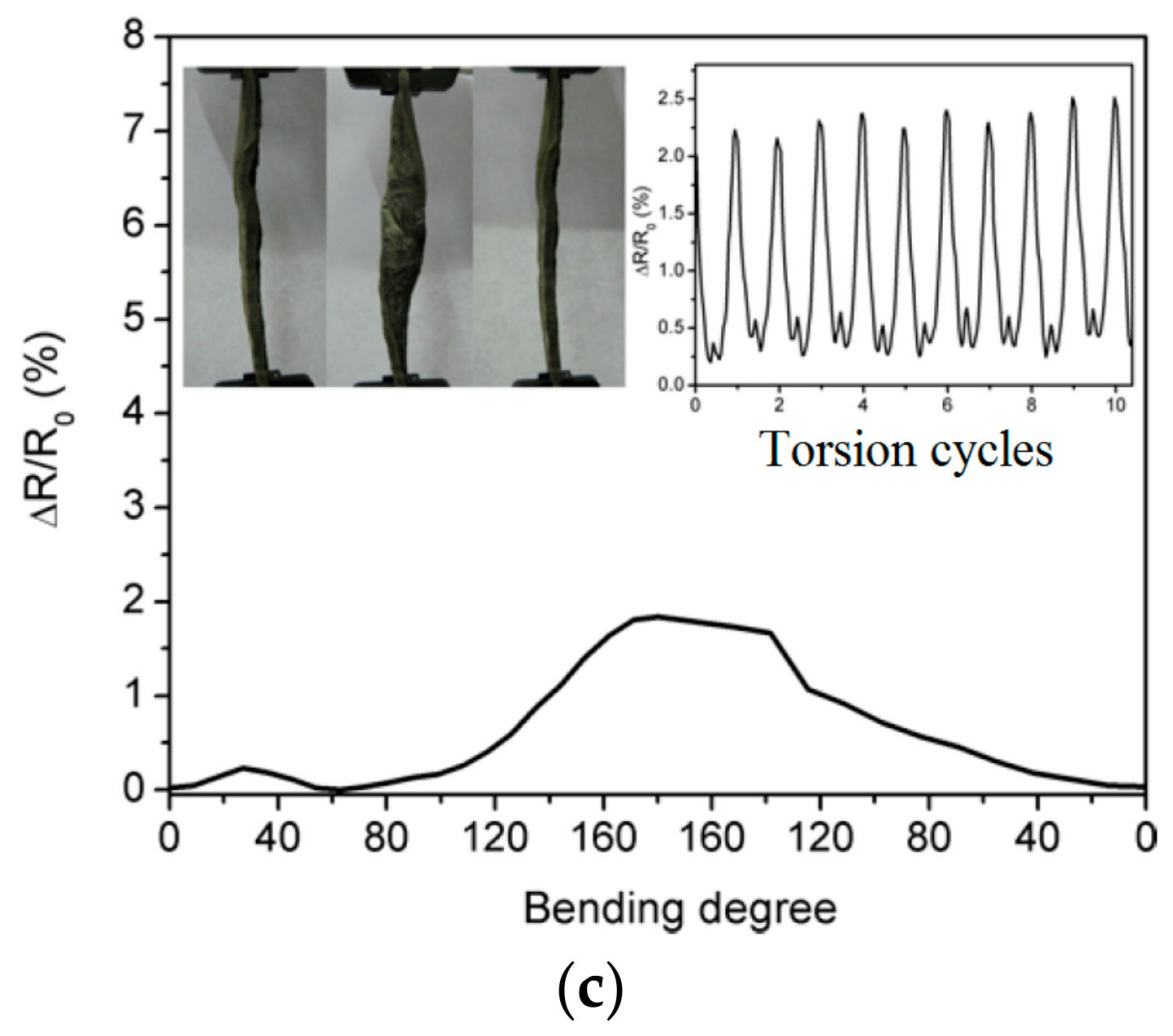
Resistance of rGO/polyimide composite under (**a**) bending, (**b**) stretching and (**c**) torsion. Reproduced with permission from [116]. Copyright 2015, American Chemical Society.

Using graphene flakes as the conducting filler and PDMS as the polymer matrix, Bang et al. [131] developed a flexible force sensor operating in the pressure range covering the entire general human pression detection range. Such graphene/PDMS nanocomposites sensors showed sensitive electromechanical response to static and dynamically applied forces, which can be used to develop force sensors capable of describing human pressure perception ability. By mixing graphene nanoflakes with PDMS, Park et al. [135] manufactured composites tunable materials properties, which can be used as smart sensors. The aspect ratio and concentration of the graphene fillers are found to have significant influences on the electromechanical behaviors of the nanocomposites (as shown in Figure 22). As seen from Figure 22a, the decrease of the aspect ratio of the graphene fillers enhance the gauge factors of the reinforced polymer composites. In addition, Figure 22b demonstrated that with the increase of the concentration of the graphene filler, the increase of the gauge factor with strain becomes less significant, whereas the linear relationship becomes more obvious. This trend agrees well with the other observations, as presented previously. Qin et al. [116] fabricated three-dimensional rGO-reinforced polyimide nanocomposite sensors. As shown in Figure 23, the nanocomposites demonstrated excellent electromechanical properties under bending, stretching and torsion deformation, and the resistance variation remained stable during each of the deformation cycles.

As presented and discussed above, various polymer matrices have been used to develop high performances with electromechanical properties. Generally, thermoplastic elastomers are ideal polymer matrices for developing flexible and stretchable piezoresistive composites [89,141].

The thermoplastic elastomers possess the elasticity, which stems from polymers, easy processability and chemical stability. Soft polymer, including PDMS and rubbers, styrene–butadiene–styrene (SBS) and their related copolymer are best options for developing piezoresistive sensors [142,143]. Particularly, among the SBS family, apart from the beneficial attributes as mentioned, SEBS has demonstrated high stretchability (i.e., deformation strain can range from less 1% to 50%), excellent endurance, elastic recovery and resistance to harsh environmental conditions [144,145,146,147].

### 3.2. Theoretical Modeling

Compared to tremendous experimental studies, relatively limited work has been found on theoretical modeling of the electromechanical behaviors of GRPCs. Apart from the fabrication of GRPC sensor, Boland et al. [117] developed a quantitative model describing the electromechanical behaviors. The authors derived the following equation to predict the electrical resistance change:

(1) $$\frac{\Delta R}{R_{0}}\approx \frac{n_{\varepsilon }}{W}{\left(\frac{2E_{mgh}}{\varepsilon_{c}^{2}Ey_{0}}\right)}^{m}$$

where *y*_0_ and *W* are the thickness and width of the sensor, *E* is the stiffness of the sensor, *m* is a mass fitted from experimental data, *ε*_c_ is the yield strain, *E_mgh_* is the energy deposited by a ball with mass *m* falling from height *h*, *n*_ε_ is a scaling exponent. It is found that the resistance change predicted by Equation (1) reasonably agrees with the experiments.

Lu et al. [148] proposed a multiscale model to study the role of interfacial debonding on the electromechanical behaviors of GRPCs. Atomistic simulation is used to model the cohesive zone. At mesoscale, the continuum mechanical model is used to capture the imperfect interfaces with the incorporation of the cohesive zone model. Using this continuum mechanical model, a representative volume element with deformation is generated to examine the effects of interfacial debonding and strain on the electrical conductivity of the GRPCs. The electrical continuum model at the mesoscale together with considering the tunneling effect is used to study the electrical conductivity. The significant electromechanical phenomenon has been observed for the composites subjected to elongation above 2%.

To further understand the electromechanical behaviors of their manufactured graphene/PDMS composites, Park et al. [135] discussed empirical models predicting the electrical and mechanical properties of the nanocomposites and addressed their limitations. The work is expected to help develop graphene-reinforced PDMS composite-based sensors for potential application in structural health monitoring. Lin et al. [136] proposed a theoretical model considering tunneling theory to interpreted their observed experimental phenomena. Using the tunneling conduction model, the experimental results can be fitted well over the 20% strain ranges. This suggests that the tunneling mechanisms plays the dominant role in the conductivity.

**Figure 24:**
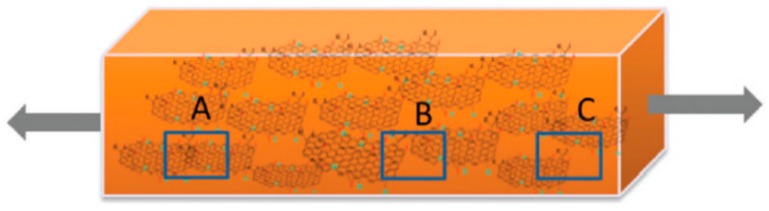
Schematic demonstration of the mechanisms electromechanical behaviors of GRPCs. Reproduced with permission from [140]. Copyright 2016. The Royal Society of Chemistry.

Tung et al. [140] presented a model as shown in Figure 24 to demonstrate the piezoresistive sensing mechanisms of GRPCs. The junction between neighboring graphene fillers can be categorized as complete connection (A), tunneling junctions (B) and disconnection (C). The total resistance of the GRPC sensor can be written as

(2) $$R_{\mathrm{total}}=R_{\mathrm{conn}}+R_{\mathrm{tunn}}+R_{\mathrm{disconn}}$$

where *R*_conn_ is the resistance of connected graphene fillers, *R*_tunn_ is the tunneling resistance between adjacent graphene fillers, and *R*_disconn_ denotes the disconnection resistance. For low strain (i.e., < 0.5%), the tunneling resistance is dominant for the increase in the total resistance. For higher strain (i.e., > 1%), the disconnection resistance dominates the sensing mechanisms.

**Figure 25:**
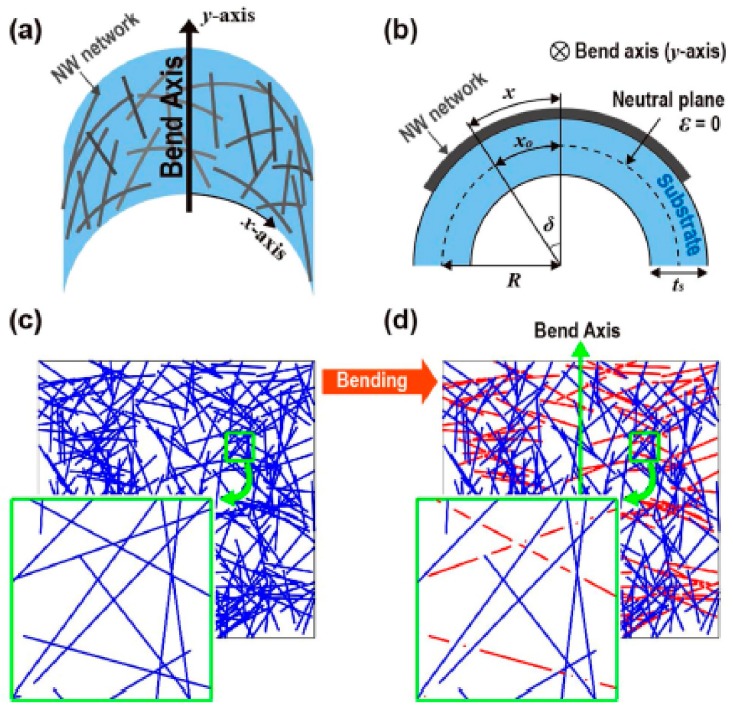
MC simulation of electrical conductivity of GRPCs under bending. (**a**) Network under outward bending; (**b**) Cross-sectional view of network under outward bending; (**c**) Network by MC simulation; (**d**) Simulation image represents network. Reproduced with permission from [149]. Copyright 2018, Springer Nature.

Monte Carlo (MC) simulation is another kind of effective method to study GRPCs nanocomposites. Hwang et al. [149] used MC simulation to develop a quantitative model predicting the electrical performance of graphene-reinforced one-dimensional composite under bending (as shown in Figure 25). Gbaguidi et al. [150] developed a 2D MC percolation network model for studying the electromechanical behaviors of carbon nanotube (CTN)- and GNP-reinforced hybrid nanocomposites. Electrical percolation and electron hopping were considered to model the filler intersection. Elastic deformation was incorporated to modify the network. The simulation results indicate that the GNPs with larger size and larger aspect ratio decrease the percolation threshold and enhance the electromechanical behaviors of the nanocomposites. The addition of GNP can enhance the electromechanical performances of the nanocomposite up to six times greater than that of the nanocomposites reinforced with CNT only. The alignment of GNPs can lead to significantly improved electromechanical behaviors of the nanocomposites.

## 4. Conclusions

Previous research on the manufacture and the electromechanical behaviors of the GRPCs is reviewed in the current paper. First the methods for producing graphene fillers are introduced with their advantages and disadvantages discussed. Five factors, including quality, cost, scalability, purity and yield, are used to evaluate the applicability and limitations for each of the methods as involved. Polymer matrices and corresponding approaches for manufacturing GRPCs are summarized and discussed. Experimental work and results on the electromechanical behaviors of GRPCs are reviewed with a focus on the change of electrical resistance/gauge factor of the GRPCs with deformation strain and displacement.

The linear relationship between the electrical resistance change and the strain is observed from the experimental data. With the increase of the graphene filler concentration, the electrical resistance change decreases and the linear relationship becomes more obvious. The gauge factor of the GRPCs increases as the strain increases, while it decreases with the increase of the graphene filler concentration. It is also found that GRPCs with a larger aspect ratio of graphene filler have a bigger gauge factor. Moreover, the cyclic test advised that the electromechanical performances varies significantly during a few cycles at the beginning. However, the electromechanical behaviors of the GRPCs become stable. GRPCs with such attributes are promising material candidates to develop smart sensors with robust performances for structural health monitoring in engineering. In addition to experiments, theoretical work on the electromechanical behaviors of GRPCs is also presented. Apart from the similar trend as observed from experimental work, the mechanisms that underpin the macroscopic electromechanical behaviors of the GRPCs are analyzed and discussed.

Compared to carbon nanotubes (CNTs), graphene fillers have demonstrated improved mechanical and physical properties, better reinforcing effects (due to their large surface area) for the polymer matrix, and moderate manufacturing cost. The GRPC-based sensors have demonstrated more stable and durable electromechanical performances compared to CNT-reinforced nanocomposites. Therefore, GRPCs can be promising material candidates for developing wearable electronics and smart sensors for structural health monitoring. Understanding the mechanisms underlying the electromechanical behavior of the GRPCs is of great essential for developing structures and devices for various engineering applications. However, compared to tremendous studies on CNT-reinforced composites and structures, relatively less work, especially theoretical modeling, has been identified on the electromechanical behaviors of GRPCs. Therefore, in the future, more efforts need to be devoted to such behaviors to promote the application of GRPCs in various engineering fields.

## Figures and Tables

**Table 1:** Comparison of methods for manufacturing graphene.

Method	Quality	Cost	Scalability	Purity	Yield
Mechanical Exfoliation	High	Low	Not applicable	Average	Low
Liquid Phase Exfoliation	High	Average	High	Average	Low
Electrochemical Exfoliation	High	High	Average	Average	Low
Chemical Vapor Deposition	High	High	Average	Average	Low
Reduction of Graphene Oxide	Low	Average	High	Low	High
Epitaxial Growth	High	Low	Low	Average	Low

**Table 2:** Polymer matrix used for graphene reinforced composites.

Graphene Type	Polymer Matrix	Preparation Method	Reference
GN, GO, rGO	PVA	Solution mixing	[23,40,79,80,81,82,83,84]
GN, GO	Polycaprolactone (PCL)	Solution mixing	[85,86]
GN	Polyurethane (PU)	Solution mixing	[21,51]
GO, GN	Polyamide (PLA)	Solution mixing	[87,88]
GO, rGO, GNP	Styrene-ethylene-butylene-styrene (SEBS)	Solution mixing	[89]
GO, GN	Polystyrene (PS)	Melt blending	[90,91]
GNP	Polyethylene terephthalate (PET)	Melt blending	[92]
GO, GN	Polypropylene (PP)	Melt blending	[93,94]
rGO	Polycarbonate (PC)	Melt blending	[95]
GN	Polymethyl methacrylate (PMMA)	In situ polymerization	[96]
GN	Polyaniline (PANI)	In situ polymerization	[97]
GN	Nylon (PA)-6	In situ polymerization	[98]
GN	Silicone	In situ polymerization	[99]
GO, GN	PS	In situ polymerization	[100,101]
GN	Polydiallyldimethylam monium chloride (PolyDDA) (PDDA)	Layer-by-layer assembly	[102]
GO	Polycyclic aromatic hydrocarbons (PAH)	Layer-by-layer assembly	[103]
GO	PVA	Layer-by-layer assembly	[104]

**Table 3:** Electromechanical behaviors of GRPCs.

Graphene Type	Polymer	Electromechanical Behaviors	Reference
GO, rGO, GNP	SEBS	The gauge factor can be as high as 120 under a 10% strain.	[89]
rGO	Elastomer	The gauge factor can reach 630 under 21.3% applied strain	[112]
GNP	PU	A stable electromechanical sensing signal can be obtained up to 90% strain.	[113]
Graphene Aerogel (GA)	Polydimethylsiloxane (PDMS)	The relative electrical resistivity change increases from 0% to 20% when the compression strain increases from 0% to 20%.	[114]
Graphene woven fabric	PDMS	Gauge factors of 103 and 106 can be obtained under strains of 6% and 7%, respectively.	[115]
rGO	Polyimide	The nanocomposites demonstrate excellent electromechanical properties under bending, stretching and torsion deformation, and the resistance variation remained stable during each deformation cycles.	[116]
GNs	Polysilicon	The electrical resistivity changes nonmonotonically with a strain and gauge factor of greater than 500 is observed.	[117]
GO	PLA/Polyethylene-glycol (PEG)	The electrical properties of the nanocomposites are sensitive to the mechanical deformations. For pressure ranges 0.6 to 8.5 MPa and 8.5 to 25 MPa, the responsivities can reach 35 mA/MPa and 19 mA/MPa, respectively.	[118]
GO	PU	The electrical resistance decreases linearly when the strain is approximately less than 60%. However, the strain further increases to be greater than 70%, and the resistance decreases exponentially. After 300 cycles at fixed strain, the electromechanical performances become stable.	[119]
rGO	PVDF	Linear fit is found for the relationship between electrical resistance and strain when the nanocomposites are subjected to deformations. The rGO-reinforced composites demonstrate the highest gauge factor among fillers as involved.	[120]
GN	PMMA	Through biaxial stretching to orientate the graphene fillers, the electrical conductivity was significantly improved in the stretching direction.	[121]
GO	PU	The electrical resistance–strain behavior is repeatable when the nanocomposites are subjected to compression cycles up to 70% strain.	[122]
GNP	Epoxy	As the graphene concentration increases, the linear growth rate of the electrical resistance change drops while the linear tendency is enhanced.	[123]
GNs	carboxymethylcellulose (CMC)	Under a compression strain of 70%, the electrical conductivity can be as high as 86.73 S/m. The gauge factor can reach 1.58 under 45%–70% compression strain.	[124]
GNs	PS	The nanocomposites demonstrate excellent electromechanical performance with sensitive electrical resistance response.	[125]
GO	PVDF	The electrical resistance change is about 27% when the nanocomposite is subjected to a strain of 10%.	[126]
GN	Epoxy	The electrical resistance changes linearly for smaller strain, and then has nonlinear, ladder-shaped growth, which indicates the irreversible deformation and damage in engineering structures.	[127]
GN	Elastomer	The electrical resistance of the nanocomposites is sensitive to the out-of-plane bending, while they are not sensitive to the in-plane stretching.	[128]
GNs	PU	When the nanocomposites are subjected to a 99% strain, the electrical resistance decreased from 5 kΩ to 25 kΩ.	[129]
rGO	PU/Polyvinyl Chloride (PVC)	The electrical resistivity of the rGO/PU and rGO/PVC composites generally decreases with the strain. However, the resistivity is almost independent on the strain with the strain range 30%–50%. The gauge factors for rGO/PU and rGO/PVC composites are observed to be 16.1 and 14.3 at 2% strain, and are 3.4 and 3.3 at 10% strain, respectively.	[130]
Graphene flakes	PDMS	The nanocomposite-based sensors showed sensitive electromechanical response to static and dynamically applied forces, which can be used to develop a force sensor capable of describing human pressure perception ability.	[131]
GN	PDMS	The nanocomposite-based sensors demonstrate high stretchability (~120%) and high sensitivity.	[132]
Graphene flakes	PDMS	The gauge factor increases with the strain for smaller graphene concentration while it keeps constant when the concentration increases to 30 wt %	[133]
Graphene foam	PDMS	With the increase of the stretching cycles, the electrical resistance first increases for the first six cycles. Then the resistance keeps constant when the strain is released.	[134]
Graphene flakes	PDMS	The aspect ratio and concentration of the graphene fillers have significant influences on the electromechanical behaviors. Graphene fillers with larger aspect ratio and great concentration are beneficial to enhance the gauge factor of the nanocomposites.	[135]
GN	rubber	The nanocomposite-based sensors exhibited a high stretchability, sensitivity (i.e., gauge factor can reach up to 82.5) and good reproducibility (up to 300 cycles) when subjected to a cyclic tensile test.	[136]
rGO	PDMS	High strain sensing sensitivity with a gauge factor of about 7.2.	[137]
GA	PDMS	The nanocomposites showed excellent electromechanical stability during a repeated compress process.	[138]
GN	PDMS	The electrical resistance change increases exponentially with pressure when the composites are under uniaxial compression. After 1000 load-release cycles, the curves remain nearly unchanged, indicating excellent durability and electromechanical stability.	[139]
GN	Epoxy	The electromechanical performance of the composites, which are subjected to static and dynamic deformation, demonstrated fast response (20 ms) and excellent sensitivity (gauge factor of 12.8).	[140]
